# Dichalcogenide and Metal Oxide Semiconductor-Based Composite to Support Plasmonic Catalysis

**DOI:** 10.1021/acsomega.2c06337

**Published:** 2023-02-13

**Authors:** Ahmed T. Alanazi, Aeshah Alotaibi, Mahdi Alqahtani, James H. Rice

**Affiliations:** † School of Physics, University College Dublin, Belfield, 4 Dublin, Ireland; ‡ King Abdulaziz City for Science and Technology (KACST), Riyadh 12371, Saudi Arabia

## Abstract

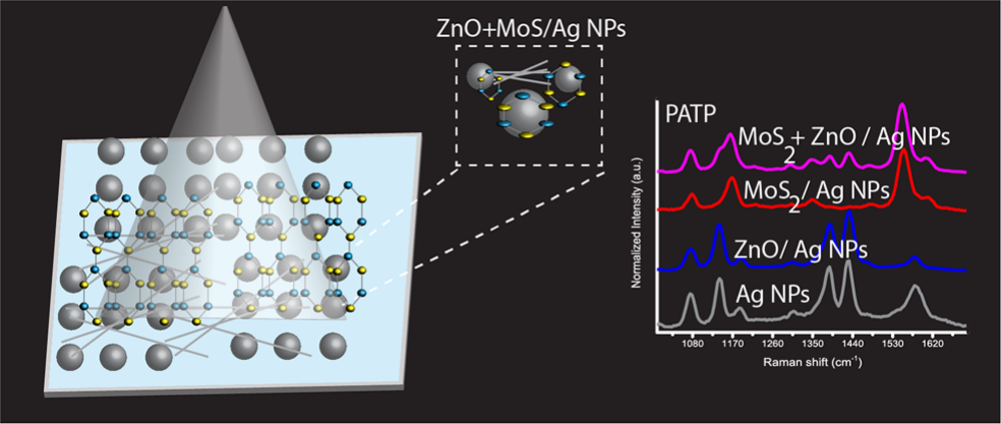

Nanocomposites comprising plasmon active metal nanostructures and semiconductors have been used to control the charge states in the metal to support catalytic activity. In this context dichalcogenides when combined with metal oxides offer the potential to control charge states in plasmonic nanomaterials. Using a model plasmonic mediated oxidation reaction *p*-amino thiophenol ↔ *p*-nitrophenol, we show that through the introduction of transition metal dichalcogenide nanomaterial, reaction outcomes can be influenced, achieved through controlling the occurrence of the reaction intermediate dimercaptoazobenzene by opening new electron transfer routes in a semiconductor-plasmonic system. This study demonstrates the ability to control plasmonic reactions by carefully controlling the choice of semiconductors.

## Introduction

Advanced materials and new techniques offer significant opportunities to advance control over surface catalytic reactions.^1−7^ Such surface reactions can be potentially applied in a wide range of areas such as chemical production or for the removal of contaminants. Currently, used catalysts have well-known limitations regarding reactivity, selectivity, and/or stability.^8−10^ For example, for many current industrial catalytic processes, catalysts require high temperatures and/or pressures to operate efficiently.^11,12^ The use of plasmon resonances in metal nanostructures to control the rate and selectivity of photocatalytic reactions offers significant potential.^13−17^ The localized surface plasmon resonance (LSPR) excitation of metal nanostructures produces enhanced light-matter interaction, resulting in a strongly enhanced plasmonic electromagnetic field on the surface of a plasmon active nanostructure.^8−12^ The oscillation of free electrons quickly decays via the excitation of energetic electron–hole pairs.^7−10^ These initially excited electrons rapidly thermalize and equilibrate via electron–electron scattering, creating a “hot” Fermi–Dirac distribution.^2−6^ Then the hot distribution cools via the coupling between the “hot electrons” and the phonons of the metal lattice. These generation “hot” electrons are seen as essential in catalysis through their interaction with the target chemical. However, hot electrons possess short subpicosecond lifetimes which presents a significant challenge for efficient surface plasmon-induced hot electron transfer catalytic reactions.^11−14^

The development of transition metal dichalcogenide (TMDC) materials has opened up new opportunities in optoelectronics applications. Molybdenum disulfide (MoS_2_) is emerging as a unique material with a range of optical and electrical properties. It demonstrates a layer-dependent band gap and excitons in both the visible and infrared regions of the electromagnetic spectrum. Among the unique characteristics of MoS_2_^18−22^ is that photogenerated excitons remain stable at room temperature because of their high binding energy. In contrast, devices made completely of MoS_2_ have a significant limitation because of their limited capacity to absorb light. MoS_2_-based heterostructures formed by combining MoS_2_ with materials such as zinc oxide (ZnO) offer the potential to enhance the optical qualities of the TMDC. ZnO possesses a wide direct bandgap of 3.37 eV at room temperature and a work function of 5.2 eV.^23,24^ In addition, ZnO is low-cost and exhibits high resistance to defects, high stability, environmentally friendly characteristics, and biosafety.^25−28^ When these plasmonic nanoparticles attach to semiconductors such as ZnO, a Schottky barrier will form at the interface between the metal and the semiconductor. The formation of this metal–semiconductor heterojunction is an effective way to enhance charge carrier separation and improve photocatalytic efficiency.^29^ Combining TMDCs nanostructures with ZnO/plasmonic metal nanomaterials offers a potential route to control surface catalytic reactions. It has been demonstrated that the addition of MoS_2_ to ZnO concentrates charge onto MoS_2_ through photoabsorption-based processes.^30,31^ This can potentially enhance plasmonic catalysis rates through a strengthened exciton–plasmon interaction between silver nanoparticles (AgNPs) and MoS_2_, creating a stronger electric field at the AgNP/MoS_2_ interface resulting in longer-lived hot electrons resulting in enhanced plasmonic catalysis properties. Additionally, MoS_2_ can protect plasmonic metals, for example, by preventing the oxidation of Ag. MoS_2_ can bind strongly to plasmonic metals such as Ag due to the favorable bonding between S (sulfide atom) and the Ag metal atoms. Simulations of AgNPs when attached to MoS_2_ showed that strong excitation-plasmon coupling of the silver lattice with MoS_2_ layers can occur,^32,33^ resulting in the altering of the density of states (DOS) and increasing the hot electrons’ lifetimes which can potentially improve plasmonic catalysis reaction rates.^34,35^ Moreover, ZnO and MoS_2_ have lattice constants that are very well matched, which makes them ideal for interfacial carrier transfer in ZnO/MoS_2_ heterostructures.^36^

Here we study the effect of combining TMDCs and metal oxides on a model plasmonic catalysis reaction. We undertake this study using the oxidation of *p*-amino thiophenol (PATP) to *p*-nitrophenol (PNTP). We show that in a model oxidation reaction PATP ↔ PNTP, the reaction is controlled by the introduction of the TMDC nanomaterial MoS_2_ to a ZnO/AgNP system by opening up new electron transfer routes. This study demonstrates the ability to control plasmonic reactions by carefully controlling the choice of semiconductor used to support the plasmon active nanomaterial.

## Results and Discussion

**Figure 1:**
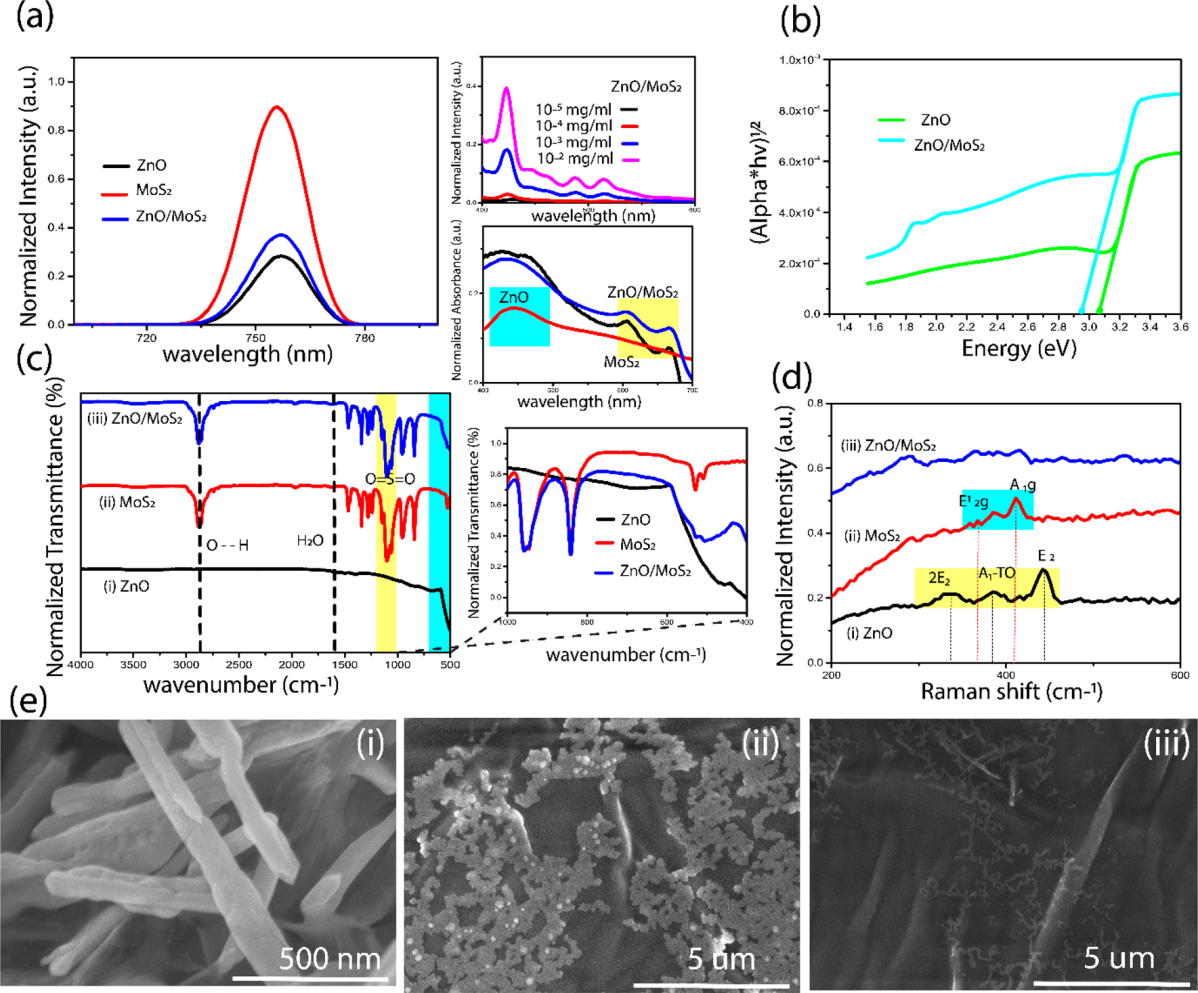
(a) Optical absorption spectra (UV–vis) of ZnO NWs (red) MoS_2_ (black) ZnO:MoS_2_ (blue). Insert: fluorescence spectra of ZnO NWs, MoS_2_, and ZnO:MoS_2_ also fluorescence spectra of ZnO:MoS_2_ at different concentrations. (b) Tauc plots of ZnO NWs and ZnO:MoS_2_ to determine the value of the band gap. (c) Raman spectra of ZnO NWs (black), MoS_2_ (red) and ZnO:MoS_2_ (blue). (d) FTIR spectra of the of ZnO NWs (black) MoS_2_ (red) ZnO:MoS_2_ (blue) (e) SEM: (i) ZnO NWs, (ii) MoS_2_ and (iii) ZnO:MoS_2_.

Photoluminescence (PL) emission spectra of ZnO and MoS_2_/ZnO mix (Figure 1a) revealed the predicted wide peak located at around 750 nm. When ZnO is introduced to MoS_2_, a significant quenching effect is noticed. This reduced PL intensity suggests that the rate of electron–hole recombination has decreased. This indicates that the TMDC and semiconductor have a strong interaction. Compared to MoS_2_ or ZnO alone, the optical characteristics of ZnO coupled with MoS_2_ exhibit no variation in absorption peak. As shown in Figure 1a, MoS_2_ exhibits an absorption peak at ca. 600 nm (arising from exciton A and B transitions), while for ZnO, the absorption peaks are found at 450 nm. After mixing the composite, we found that the MOS_2_/ZnO mix exhibits stronger absorption ability than pristine MoS_2_, suggesting that the heterostructure has more intensive light-matter interaction.^37,26,29,58^ In Figure 1b, a graph depicting the relationship between the photon energy *h*ν (eV) and (ν*hν) 1/*n* was plotted, where *n* is a constant that relates to various electronic transition types (*n* = 3 for indirect forbidden transitions, *n* = 2 for indirect allowed, *n* = 3/2 for direct forbidden, and *n* = 1/2 for direct allowed).
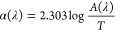
1where α stands for the absorbance coefficient, *A* represents the absorbance, and *T* represents the sample’s thickness.^26,38−50^ A linear fit to this data reveals that the absorption band edge shifts to the red shift by about 0.2 eV from 3.1 eV for ZnO to 2.9 eV for ZnO/MoS_2_. This redshift possibly occurs from an increase in AgNPs electron density which changes the reflective index of the nanoparticles. Studies of MOS_2_/ZnO using Fourier transform infrared (FTIR) spectroscopy were conducted (Figure 1c). The characteristic stretching mode of the ZnO bond is assigned a large vibration band in the FTIR spectra ranging from 400 to 550 cm^–1^. The presence of hydroxyl is shown by a broad peak at 3430 cm^–1^ stretching mode and 1330 cm^–1^ to 1670 cm^–1^ bending mode.^59,60^ In addition, a band at 490 cm^–1^ is observed corresponding to a Mo–S vibration. Raman spectra of ZnO in (Figure 1d) showed the strongest peak at 440 cm^–1^ which is attributed to the phonon mode wurtzite hexagonal phase E_2_ of ZnO. In addition, two peaks are seen at 330 and 380 cm^–1^, which are allocated to the multiphoton process 2E_2_ and A1-TO modes, respectively. In addition to the Raman spectra of MoS_2_ excited at 532 nm, we noted the two main phonon peaks located at 370 cm^–1^ arising from the E^1^_2g_ in-plane and 410 cm^–1^ assigned to A_1g_ out-of-plane.^51,37^ The Raman spectra for MoS_2_ and ZnO combined showed spectral features arising from combining the Raman spectral features from each component. Scanning electron microscopy (SEM) images of the pure MoS_2_, ZnO and MoS_2_/ZnO combined are shown in (Figure 1e) respectively. The pristine MoS_2_ nanoparticles have a size range between 50 and 1000 nm and are clustered. MoS_2_:ZnO nanocomposites, the MoS_2_ layers whose crystalline sizes are ca. 300 nm, are self-restacked and form thick layers. The small panels of ZnO are decorated on the surface, and edge of the large, in a small number of layered MoS_2_.

**Figure 2:**
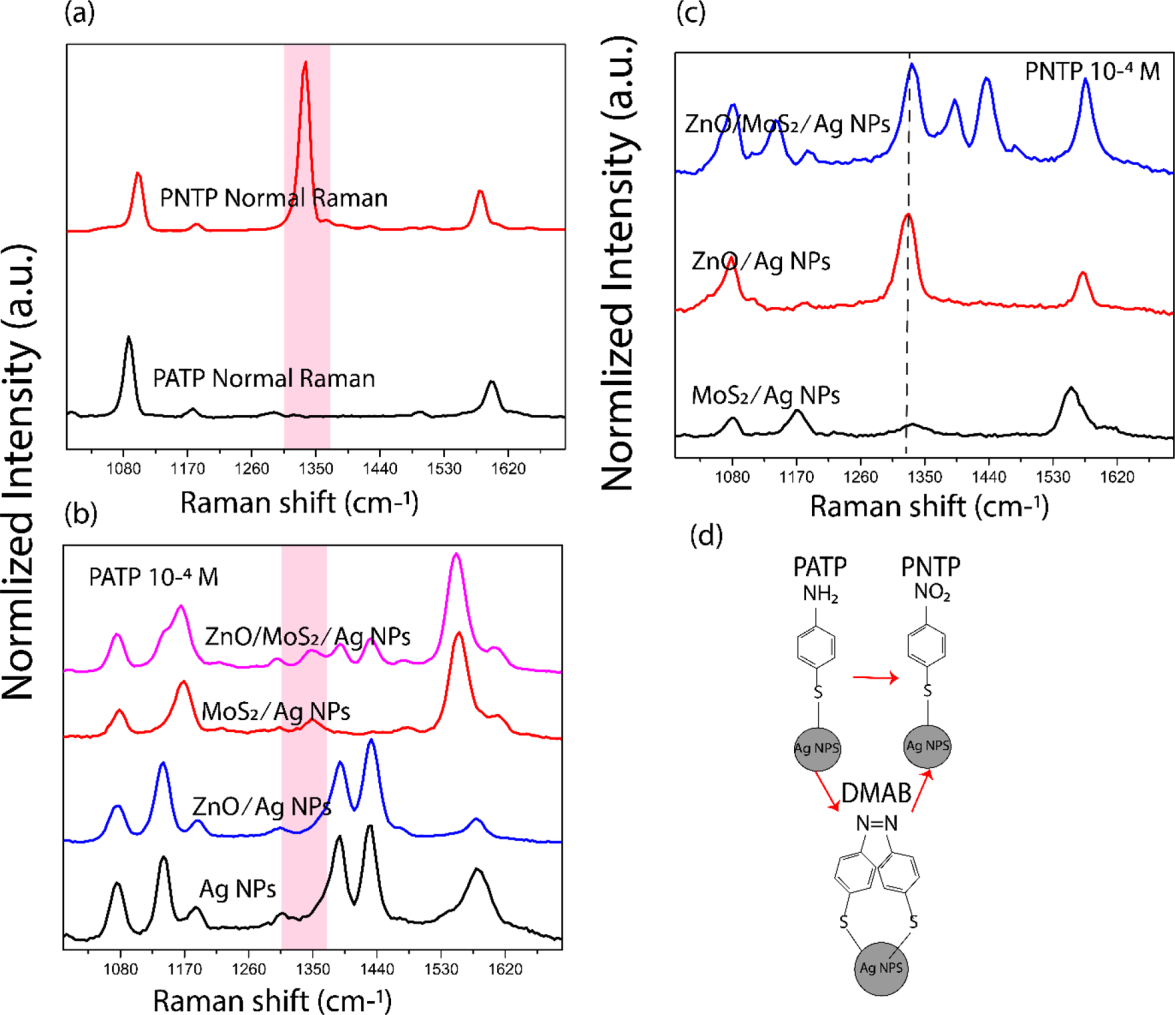
(a) Normalized Raman spectra for PATP or PNTP on a dielectric substrate. (b) Normalized SERS spectra, PATP recorded on AgNPs only (black), ZnO/AgNPs (blue), MoS_2_/AgNPs (red) and the composite of ZnO:MoS_2_ /AgNPs (Pink). (c) Normalized SERS spectra of PNTP deposited on ZnO/AgNPs, MoS_2_/AgNPs and ZnO/MoS_2_ /AgNPs (d) Schematic of the oxidation reaction of PATP showing the formation of DMAB and PNTP.

An investigation into the effect that MoS_2_/ZnO had on the plasmonic catalytic conversion of PATP to PNTP was undertaken. The Raman spectra of PATP and PNTP powder were first acquired using a dielectric substrate (Figure 2a). The Raman spectra shows A_1_ modes with peaks at 1080 and 1595 cm^–1^ in agreement with literature values.^2,3,8−14^ The SERS spectrum of PATP on AgNPs (Figure 2b) reveals strong A_1_ 1077, 1190, and 1600 cm^–1^ in addition to b_2_ modes at 1142 ascribed to C–H bend vibration, 1391 and 1440 cm^–1^ ascribed to the stretching vibration, and 1550 cm^–1^ for both frequency location and relative intensities.^8−14^ It has been determined that a photocatalytic process takes place on the metal substrate, which is responsible for this difference in spectra when comparing PATP on a dielectric substrate to AgNPs. This photocatalytic process may result from the hot electrons formed when the Raman excitation laser excites the localized surface plasmon resonance (LSPR) of AgNPs.^1−3,8−14^ There are two possible mechanisms for this plasmon-driven oxidation of PATP to *p*,*p*′-dimercaptoazobenzene (DMAB). First, the hot electrons that are produced as a result of plasmon decay are transferred to adsorbed singlet oxygen molecules from the surrounding air. This produces reactive triplet ^3^O_2_, which is then engaged in the oxidation of PATP to DMAB. The second mechanism is that plasmonic hot electrons leap off the surface of the metal, and the hot holes that are left behind on the metal oxidize PATP to DMAB. It has been found that if a sufficiently enough external stimulus was introduced into the system, PATP would be oxidized to PNTP rather than oxidized to DMAB.^52,8−14^ PATP on AgNPs/ZnO (Figure 2b) produces a Raman spectrum that replicates for PATP on only AgNPs with the spectrum assigned to DMAB. In contrast, when PATP is present on AgNPs/MoS_2_, the substrate prevents the formation of DMAB. The SERS spectrum possesses a peak at 1350 cm^–1^ arising from the presence of PNTP formed from the oxidation of PATP. When PATP is added to AgNPs/MoS_2_/ZnO, the spectra show features arising from a combination of DMAB and PNTP in comparison to when AgNPs/ZnO is studied where DMAB only is observed. Examining placing PNTP on the semiconductor-plasmonic substrate was then undertaken to assess how adding MoS_2_ to ZnO/AgNPs affects this molecule. For PNTP on AgNP/ZnO, the Raman spectra of PNTP is preserved, with the Raman spectra (Figure 2c) showing the same features as recorded for PNTP (Figure 2a). In contrast, when MoS_2_ is added forming ZnO/MoS_2_/AgNP, PNTP partially converts to DMAB with the Raman spectra showing peaks assigned to PNTP and DMAB.^9−14,52^

**Figure 3:**
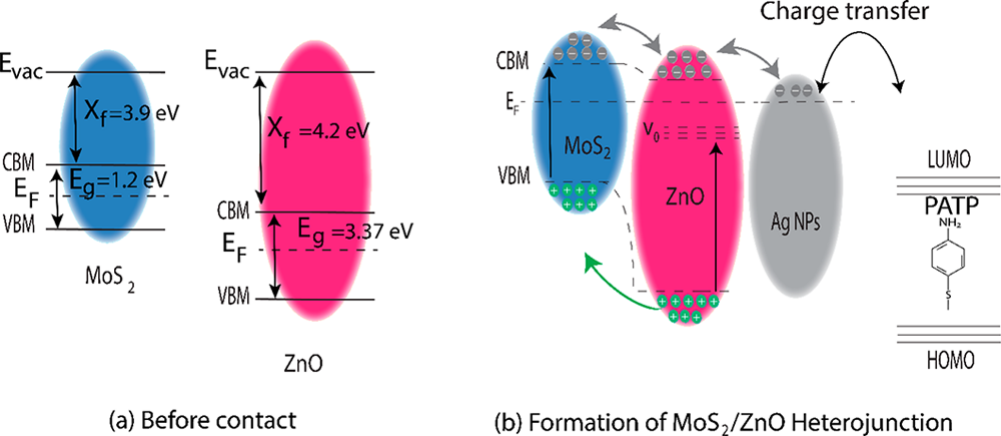
An energy band diagram showing the electronic transition between ZnO:MoS_2_ and AgNPs and the PATP analyte molecule. (a) MoS_2_/ZnO before contact and (b) formation of MoS_2_/ZnO heterojunction.

A band diagram (Figure 3a) shows a band diagram of ZnO and MoS_2_. The semiconductor ZnO has an electron affinity (χ) = 3.87 eV, work function (φ) = 5.28 eV, and bandgap (*E*_g_) = 3.26 eV.^29,53,54^ While, MoS_2_ has an estimated *E*_g_ = 1.4 eV, φ = 5.15 eV, and χ = 4.3 eV.^25,38,55^ When adding AgNPs to MoS_2_ and ZnO, a heterojunction is formed (Figure 3b). Following the application of the Raman excitation wavelength (532 nm) electron–hole pairs are formed in MoS_2_. Electrons from MoS_2_ conduction band can transfer the Ag Fermi level. As mentioned earlier, the transfer of electrons from MoS_2_ conduction band to the Ag Fermi level will suppress PL (Figure 1a), as the Schottky contact will reduce the rate of radiative recombination. When ZnO is introduced, this semiconductor forms a ZnO/MoS_2_/AgNP system. Photogenerated electrons from MoS_2_ could transfer to ZnO, and as the conduction band edge potential of ZnO is lower in energy than MoS_2_, the electrons in the conduction band of MoS_2_ could transfer into the conduction band of ZnO.^29,38,51,53,54,56^ This results in reduced efficiency of forming PNTP from PATP for ZnO/MoS_2_ /AgNPs relative to MoS_2_/AgNPs (as observed in Figure 2).

Overall, we demonstrate that dichalcogenides when combined with metal oxides offer the potential to control charge states in plasmonic nanomaterials. This is demonstrated in a model oxidation reaction PATP ↔ PNTP.^57^ The use of molybdenum disulfide influences the reaction of intermediate dimercaptoazobenzene by introducing a new electron transfer pathway thereby affecting reaction selectivity. The use of heterostructures potentially offers a route for the regulation of the reaction pathway and reduction of consecutive reactions which may be used to reduce overoxidation.

## Materials and Methods

### Substrates and Chemicals

Transition metal dichalcogenides (TMDCs) molybdenum disulfide suspension (MoS_2_ lot # MKCH5329) liquid, 5 mg/mL in H_2_O, 50–1000 nm thickness, 3 layers, (Aldrich 902012-25 ML) 2D single layered nanomaterials were used. Zinc oxide nanowires (ZnO NWs) (Aldrich: SKU: 774006–500MG, CAS: 1314-13-2, MW: 81.39), with a length of 4–5 m, were placed into distilled water at a concentration of 10^–1^ M and sonicated for 30 min with an ultrasonic cleaning bath to make sure the wires were spread out evenly in the water. The solution was then casted over a coverslip or silicon substrate to produce zinc oxide nanowire substrates. Nanocomposite ZnO-NWs/MoS_2_/AgNPs substrates were produced by combining ZnO, MoS_2_ AgNPs (Aldrich: SKU 730807-25 ML; nanoparticles, 40 nm particle size (TEM), 0.02 mg/mL in an aqueous buffer) solutions in a 1:1:1 ratio after depositing the mixture on a coverslip or a silicon substrate.

### Preparing Solutions of Probe Molecules

A solution of 4-aminothiophenol (4-ABT) in methanol at a concentration of 10^–2^ M was produced. The solution was then diluted to a final concentration of 10^–5^ M using deionized water. Then, 30 L of the liquid probe molecule was drop cast onto the substrates prior to the Raman observations. 1:1:1 ratio (semiconductors:metals:molecule).

### Raman Spectroscopy

SERS spectra are collected using a monochromatic light green laser (HeNe, ThorLabs). Excitation wavelength is 532 (nm). Laser power and energy meter: a microscopy slide power meter sensor head (SN: 09113026, S121 C, 400–1100 (nm), 500 (mW), LMR1/M, Thorlabs) and energy meter are used to measure the incident laser power. The energy of the laser power is focused by an attenuator at 5 mW to control the laser power at a distance of ca. 2 cm for the entire experiment. Briefly, the beam passes through an interference filter and is directed by a mirror to angle prism, which drives the beam at 90 towered the sample. Then, it passes through a lens, which can be focused onto the samples to obtain the best signals. Here, the sample is excited and scatters light, which is collected by the lens and passes through a notch filter. This lowers the impact from the laser line before it enters the spectrograph. Raman spectra are collected with an exposure time of 1 s and 10 accumulation modes. Calibration of the Raman spectrographic windows is conducted by acquiring a Raman spectrum from the toluene and using it as a standard spectrum. The mean and standard deviation of 10 measurements is recorded.

### Optical Spectroscopy UV–vis Absorption

Optical absorbance (UV–vis) measurements were accomplished with the use of an absorbance spectrometer (V-650, JASCO, Inc.), with the following settings: 1 nm step size, UV–vis bandwidth of 2 nm, and 200 nm/min scan speed across a range of 200–800 nm. For the purpose of performing out the measurements, a coverslip substrate was used.

### Fourier Transform Infrared Spectroscopy

Setup for Fourier transform infrared spectroscopy (FTIR) measurement parameters included a resolution of 4 cm^–1^, a sample scan time of 8 scans, a measurement period of more than 10 s, data stored between 400 and 4000 cm^–1^, result spectrum transmission mode, and accessory ATR platinum diamond. As a solid state, we recorded the FTIR spectra of both ZnO and MoS_2_ as well as the composite of ZnO and MoS_2_. The Alpha Platinum Bruker system was used in order to acquire data from the FTIR instrument.

### Transmission Electron Microscopy

TEM is used to examine the thin sample ultrastructure (limited by the penetration of electron beam). The transmission electron microscope utilizes an electromagnetic lens to concentrate electrons into a very tiny beam. The electrons then either scatter or strike a fluorescent screen at the bottom of the microscope after passing through a very thin object. An picture of the specimen with its many components shown in various hues based on its density shows on the screen. This picture may then be examined or photographed immediately inside the TEM.

## Data Availability

The data that supports the findings of this study are available within the article.
